# The effects and potential applications of concentrated growth factor in dentin–pulp complex regeneration

**DOI:** 10.1186/s13287-021-02446-y

**Published:** 2021-06-19

**Authors:** Zixia Li, Liu Liu, Liu Wang, Dongzhe Song

**Affiliations:** State Key Laboratory of Oral Diseases & National Clinical Research Center for Oral Diseases & Department of Cariology and Endodontics, West China Hospital of Stomatology, Sichuan University, 14# Third Section, Renmin Nan Road, Chengdu, 610041 China

**Keywords:** Concentrated growth factor, Dentin–pulp complex, Stem cell, Regenerative endodontic treatment

## Abstract

The dentin–pulp complex is essential for the long-term integrity and viability of teeth but it is susceptible to damage caused by external factors. Because traditional approaches for preserving the dentin–pulp complex have various limitations, there is a need for novel methods for dentin–pulp complex reconstruction. The development of stem cell-based tissue engineering has given rise to the possibility of combining dental stem cells with a tissue-reparative microenvironment to promote dentin–pulp complex regeneration. Concentrated growth factor, a platelet concentrate, is a promising scaffold for the treatment of dentin–pulp complex disorders. Given its characteristics of autogenesis, convenience, usability, and biodegradability, concentrated growth factor has gained popularity in medical and dental fields for repairing bone defects and promoting soft-tissue healing. Numerous in vitro studies have demonstrated that concentrated growth factor can promote the proliferation and migration of dental stem cells. Here, we review the current state of knowledge on the effects of concentrated growth factor on stem cells and its potential applications in dentin–pulp complex regeneration.

## Dentin–pulp complex preservation

Dental pulp, the only soft tissue in teeth, consists of fibroblasts, odontoblasts, immune cells, nerves, blood vessels, extracellular matrix (ECM), interstitial fluid, and other cellular components and is responsible for nourishing teeth, forming dentin, transmitting sensory information, and providing immunoprotection. Dentin is a highly calcified and inextensible tissue under the enamel and cementum surrounding the dental pulp, forming the pulp cavity. Dentin and dental pulp, which form the dentin–pulp complex (DPC), originate from the tooth germ’s dental papilla during embryogenesis and have interrelated functions [1]. Odontoblasts in dental pulp produce tertiary dentin upon physiological or pathological stimulation including by pathogens, thereby serving a defence function [2]. Thus, a healthy DPC maintains the structural integrity and normal function of teeth.

The DPC is susceptible to external stimulation including infection (e.g., periodontitis) and trauma [3]. As an anatomical feature of the pulp chamber, the dental pulp has poor collateral vascularisation; upon infection, inflammatory products in the pulp chamber cannot be rapidly excreted, which increases internal pressure in the pulp cavity and leads to the spread of inflammation and irreversible pulpitis, which requires root canal treatment (RCT). This involves the complete removal of the pulp tissue followed by mechanical debridement and chemical disinfection and obturation of the root canal with filler materials. Although RCT can effectively control infection and preserve dentin to a certain extent, it has several drawbacks. The loss of dental pulp causes endodontically treated teeth to become devitalised, brittle, discoloured, and susceptible to postoperative fracture [4]. Furthermore, with the loss of pulp in immature permanent teeth, dentin formation, root development, apical closure, and subsequent tooth maturation cease [5]; this eventually affects the lifespan of teeth, leading to a decline in patients’ oral health. Therefore, the effective preservation of injured dental pulp is a primary goal of dental treatment.

Regenerative endodontic treatment based on tissue engineering offers an alternative to root canal therapy by preserving or replacing the DPC, facilitated by the development of new biomaterials and stem cell (SC) technologies [6]. In tissue engineering, three core components—namely, SCs, scaffolds, and growth factors (GFs)—are regulated in time and space to regenerate tissue [7]. The characteristics of scaffolds influence SCs and tissue regeneration; criteria for an ideal scaffold include chemical and physical stability, biocompatibility, adhesion, controlled degradation, and mechanical strength [8]. Moreover, as an artificial carrier, the scaffold should contain GFs that promote cell proliferation and differentiation [9]. Various biomaterials have been developed as scaffolds for the regeneration of dental pulp based on natural or synthetic polymers, bioceramics, and hydrogels as well as platelet concentrates such as platelet-rich plasma (PRP), platelet-rich fibrin (PRF), and concentrated (C)GF, which contain many GFs and have been used in clinical practice to promote healing of hard and soft tissues [10]. CGF—which does not contain bovine thrombin and anticoagulants—overcomes the shortcomings in preparation and the high cost of PRP and PRF and is considered as an ideal biomaterial as it releases GFs that promote cell proliferation and differentiation as a component of the three-dimensional network supporting the organisation and vascularisation of regenerated cells. Therefore, CGF is considered an ideal biological material, which can address the limitations of traditional treatment methods in DPC regeneration.

With the increasing interest in DPC regeneration, many researchers have studied the basic and clinical effects of CGF in DPC regeneration. Currently, published review articles have discussed the use of CGF in periodontal regeneration, facial reconstruction, and dental implants. Still, no review article has summarised the role of CGF in DPC regeneration; therefore, our narrative review aims to examine the scientific evidence regarding the use of CGF in DPC regeneration to give reliable and useful information for clinical work and guidance for future research. This review is intended to elucidate the in vitro biological effects of CGF on SCs involved in DPC regeneration and the clinical application progress of CGF in DPC regeneration.

## Physiological and biological characteristics of CGF

Platelet concentrate containing multiple autogenous GFs and a fibrin scaffold was discovered in 1974 and contributed to the advancement of regenerative medicine [11]. PRP, a first-generation platelet concentrate, has been used in various medical applications. However, its use has been restricted in recent years because the thrombin and calcium chloride added to enhance fibre polymerisation were shown to cause adverse effects such as cross-infection and immune rejection [12]. To overcome these problems, PRF was developed by performing a centrifugation step without adding anticoagulant [13]. Unlike PRP, the fibrin matrix of PRF acts as a 3D scaffold that allows the slow release of GFs while providing a space for cell adhesion, migration, and differentiation [14]. In 2006, Sacco and colleagues developed CGF from PRF by changing the centrifugation speed, which induced the transformation of fibrinogen into fibrin that can form a matrix with high tensile strength and promoted platelet rupture and GF release [15, 16]. Thus, CGF is superior to PRP and PRF in terms of composition and clinical applicability.

**Fig. 1:**
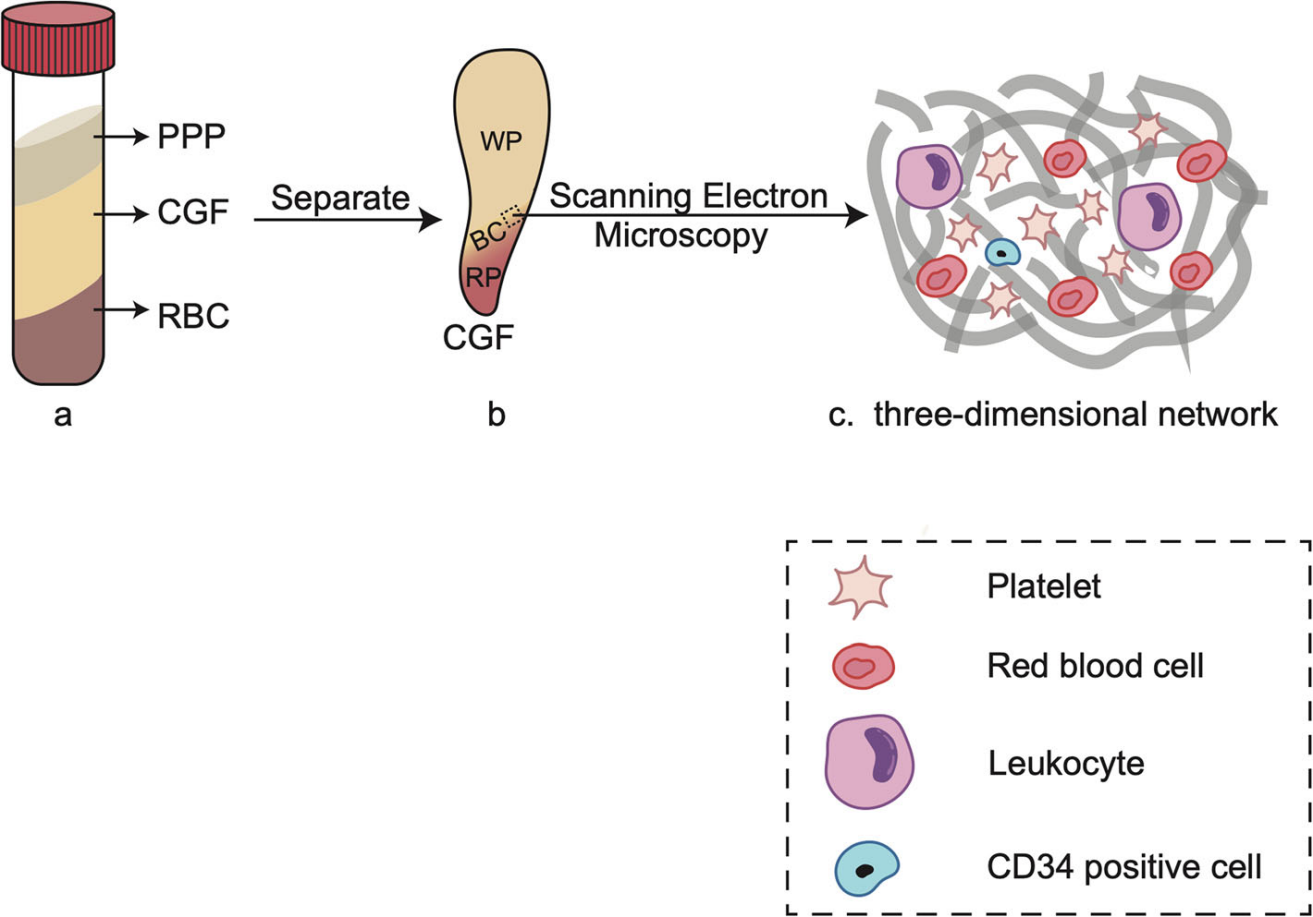
The histological and morphological observation of the CGF. **a** The blood samples after centrifugation yield a three-layer product consisting of the upper plaletet poor plasma(PPP) and lower red blood cell (RBC) layers with concentrated growth factor (CGF) gel in the middle. **b** The concentrated growth factor (CGF) gel is divided into 3 parts: the upper white part (WP) and lower red portions (RP) with the buffy coat (BC) in the middle. **c** The ultrastructure of the CGF (scanning electron microscopy observation): numerous cellular components including platelets, red blood cells leucocytes, and CD34-positive cells are embedded in the three-dimensional network

In the preparation of CGF, blood samples are processed by programmed centrifugation, yielding a three-layer product consisting of the upper plaletet poor plasma(PPP) and lower red blood cell (RBC) layers separated by the CGF gel, which also has three fractions—namely, the upper white part (WP) and lower red portions (RP) with the buffy coat (BC) in the middle [17] (Fig. 1 (a, b)). Scanning electron microscopy examination of CGF has revealed that the upper portion is a 3D network predominantly composed of fibrin with a few small-diameter fibrillin molecules similar to natural fibrin and favours cell adhesion. Meanwhile, the lower portion contains numerous cellular components including platelets, leucocytes, and RBCs [18]; notably, a large number of cluster of differentiation (CD)34-positive cells—which are involved in angiogenesis—are also present [19] (Fig. 1 (c)).

Activation of platelets packed in the fibrin scaffold of CGF through fibrinolysis can lead to the continuous release of GFs such as transforming growth factor (TGF)-β1, platelet-derived growth factor (PDGF)-BB, insulin-like growth factor (IGF)-1, bone morphogenetic protein (BMP), vascular endothelial growth factor (VEGF), epidermal growth factor (EGF), and basic fibroblast growth factor (bFGF), which are required for the regulation of SC activity in tissue engineering [15, 20]. TGF-β1 is a chemotactic and mitogenic factor in most physiological contexts that promotes mesenchymal stem cells(MSCs) proliferation and ECM synthesis [21]. PDGF-BB stimulates the proliferation of fibroblasts, osteoblasts, and MSCs and also participates in angiogenesis and collagen biosynthesis [22]. IGF-1 regulates the proliferation, migration, and differentiation of multiple cell types and induces peripheral nerve formation [23]. VEGF is a key regulator of endothelial cell proliferation and migration in angiogenesis and modulates vascular permeability in an ischemic environment during neovascularisation [24]. BMPs are a family of secreted multifunctional proteins involved in bone formation and development [25]. EGF is a 53-amino acid peptide with roles in cell differentiation, migration, and apoptosis and also acts as a potent mitogen in vitro and in vivo [26].. bFGF, a single-chain protein with mitogenic and angiogenic activities, promotes the repair of damaged endothelial cells and angiogenesis [27]. Table 1 summarises the main bioactive GFs released by activated platelets in CGF and their potential functions on SCs.

**Table 1:** Main bioactive growth factors released by activated platelets in CGF and their potential functions on SCs

Name	General function	Potential functions on SCs
Transforming growth factor (TGF)-β1	A chemotactic and mitogenic factor	It promotes MSCs proliferation and ECM synthesis. It also is effective in the odontoblastic differentiation of MSCs [28].
Platelet-derived growth factor (PDGF)-BB	A chemotactic and mitogenic factor	It promotes the homing of MSCs. It stimulates MSCs, resulting in enhanced angiogenesis and osteogenesis with a dose-dependent effect [29].
Insulin-like growth factor (IGF)-1	Regulating the proliferation, migration, and differentiation of multiple cell types	It promotes osteogenic proliferation and differentiation of DPSCs and SCAPs and promotes alkaline phosphatase production [30, 31].
Vascular endothelial growth factor (VEGF)	A key regulator of endothelial cell proliferation and migration in angiogenesis	It improves the proangiogenic capability of DPSCs and PDLSCs through accelerating the differentiation of SCs into endothelial cells.
Bone morphogenetic protein (BMP)	A family of secreted multifunctional proteins involved in bone formation and development	It promotes osteogenic/odontogenic differentiation of SCs from different dental tissue sources. It also shows potential in inducing new bone formation and promoting the terminal differentiation of odontoblasts.
Epidermal growth factor (EGF)	A 53-amino acid peptide with roles in cell differentiation, migration, and apoptosis	It stimulates the osteogenic potential of DPSCs [32] and promotes BMSCs proliferation and migration.
Basic fibroblast growth factor (bFGF)	A single-chain protein with mitogenic and angiogenic activities	It, as an effective homing/migration factor, promotes the migration of DPSCs. It also inhibits mineralisation and promotes neuronal differentiation of DPSCs [33].

*Abbreviations*: *SCs* stem cells, *DPSCs* dental pulp stem cells, *SCAPs* stem cells of the apical papilla, *PDLSCs* stem cells of the periodontal ligament, *BMSCs* bone marrow-derived mesenchymal stem cells, *MSCs* mesenchymal stem cells

## Materials and methods

The PubMed, MEDLINE, and Cochrane databases were searched from January 2000 to December 2020 to find published studies on the in vitro and clinical effects of CGF in DPC regeneration. The papers were limited to those published in the English language only, and the keywords used were as follows: “concentrated growth factor” (OR “CGF”), AND “stem cells” OR “cells” OR “cell proliferation” OR “cell migration” OR “cell differentiation”, AND “pulp regeneration” OR “regenerative endodontic treatment” OR “vital pulp therapy”. Articles irrelevant to the topics and repetitive in content were excluded. All authors discussed and agreed which articles met the inclusion criteria and which articles should be excluded. The full texts of all corresponding articles were assessed, and 11 articles were included in this review.

## Effects of CGF on SCs in DPC regeneration

**Fig. 2:**
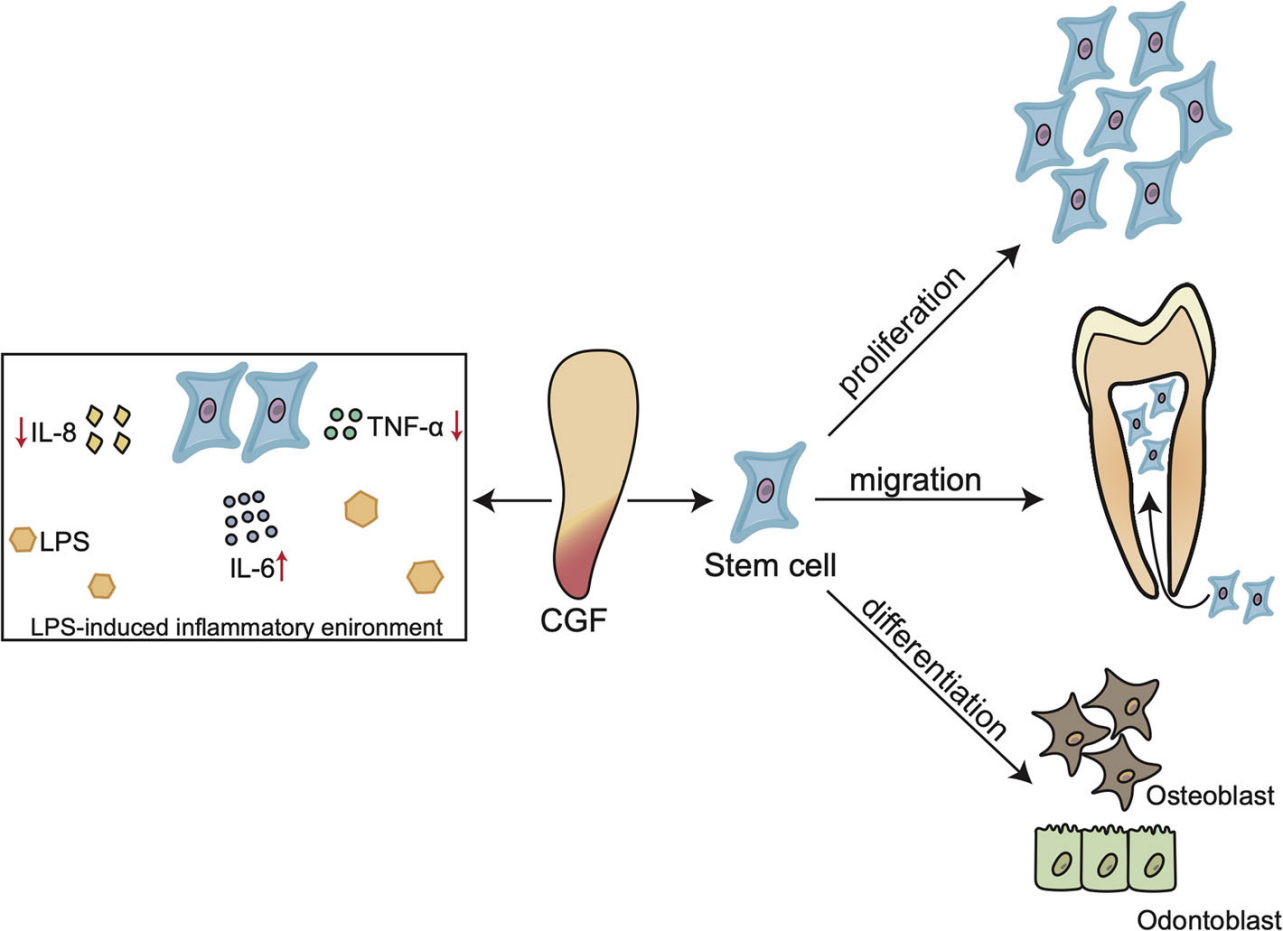
Effects of CGF on SCs in DPC regeneration. The left part shows that CGF can regulate the lipopolysaccharide (LPS)-induced inflammatory response in stem cells by inhibiting the expression of the proinflammatory cytokines IL-8 and TNF-α but not IL-6. The right part shows that CGF can promote the proliferation, migration, and osteogenic/odontoblastic differentiation of stem cells

SCs related to DPC regeneration were used in 10 studies to evaluate their proliferation, migration, and differentiation under treatment with CGF (Table 2). DPC regeneration is a complex process involving cell proliferation, migration, and differentiation; dentin ECM remodelling; and angiogenesis [43]. SCs are undifferentiated clonogenic cells that continuously undergo self-renewal and differentiation [44]. A variety of SCs involved in DPC regeneration have been isolated from dental tissue including dental pulp stem cells (DPSCs), SCs of the apical papilla (SCAPs), periodontal ligament stem cells (PDLSCs), and bone marrow-derived mesenchymal stem cells (BMSCs) [45, 46]. GFs activate multiple signalling pathways and mechanisms that regulate the behaviour of SCs by binding to cell surface receptors [47]. BMP, TGF-β1, FGF, PDGF-BB, and IGF-1 among others are key GFs involved in DPC regeneration [48]; given their presence in CGF, 10 studies have investigated the effect of CGF on SCs in vitro in order to evaluate its potential to induce DPC regeneration (Fig. 2).

**Table 2:** The effects of CGF on SCS regeneration in DPC regeneration and its potential molecular mechanism

Authors (year)	Stem cells	Type of evaluation	Methods	Main result	Potential mechanism
Hong et al. (2019) [18]	h-SCAPs	Proliferation, migration, and odonto/osteogenic differentiation	Cell counting kit-8; Transwell Filter Inserts; ARS and qPCR (ALP, DSPP, DMP-1)	CGF can significantly promote the proliferation, migration, and differentiation of SCAPs, and no dose-dependent manner effect.	More migration effect may be caused by the abundant chemotactic factors released from the CGF, including PDGF-BB and bFGF.
Hong et al. (2018) [34]	h-SCAPs	Proliferation, migration, and odonto/osteogenic differentiation	Cell counting kit-8; Transwell assays; ARS and qPCR (ALP, DSPP, DMP-1)	CGF can significantly promote the proliferation, migration, and differentiation of SCAPs, and no dose-dependent manner effect. CGF had an early inhibitory effect on the odonto/osteogenic differentiation of SCAPs.	The early inhibitory effect may be caused by proinflammatory factors such as TNF-α and IL-1 in CGF.
Xu et al. (2019) [35]	h-DPSCs exposed to LPS	Proliferation, migration, and odonto/osteogenic differentiation	Cell counting kit-8; Transwell assays; ALP activity, ARS, and qPCR (DMP-1, DSPP, OPN, RUNX2)	CGF promoted the proliferation, migration, and differentiation of DPSCs exposed to LPS in a dose-dependent manner.	The secretion of TNF-α and IL-8 in DPSCs treated by CGF could promote the DPSCs migration.
Tian et al. (2019) [36]	h-DPSCs	Proliferation, migration, and odonto/osteogenic differentiation	Cell counting kit-8; Transwell assays; ALP activity, ARS, and qPCR (DMP-1, DSPP, BSP, RUNX2)	CGF promoted the proliferation and migration of DPSCs in a dose-dependent manner, and CGF enhanced DPSCs odonto/osteogenic differentiation by upregulating RUNX2 transcription.	BMP-2/SMAD5/Runx2 signaling axis is related to CGF-mediated DPSCs mineralization.
Jin et al. (2018) [37]	h-DPSCs	Proliferation, migration, endothelial differentiation, and odontoblastic differentiation	Cell counting kit-8; Scratches; ALP activity, ARS, western blotting (VEGFR2, CD31), qRT-PCR (DMP-1, DSPP), and tube formation assay	CGF promoted the proliferation of DPSCs in a dose-dependent manner, and high concentrations of CGF inhibited the endothelial differentiation and odontoblastic differentiation of DPSCs.	The negative role of high-dosage CGF may be associated with the excess content of TGF-β, IL-1β, and IL-6 with increasing concentration.
Aghamohamadi et al. (2020) [38]	h-PDLSCs	Proliferation	MTT assay	CGF promoted PDLSCs proliferation in no dose-dependent manner, and high concentrations of CGF markedly inhibited the proliferation of PDLSCs	The high-dosage inhibition effect is thought to be mediated by TGF-β and proteolytic enzymes.
Li et al. (2019) [39]	h-PDLCs stimulated by TNF-α	Proliferation, osteogenic differentiation	Cell counting kit-8 assays; ARS, ALP activity, western blotting, and qPCR (OCN, OSX, RUNX2)	CGF enhanced h-PDLCs proliferation and osteogenic differentiation in the presence of TNF-α-induced inflammation.	TGF-β1 contained in CGF relieved the inhibitory effect of TNF-α on the osteogenic differentiation of h-PDLCs by inducing the upregulation of Runx2 transcription.
Yu and Wang (2014) [40]	Beagle-PDLSCs	Proliferation, osteogenic differentiation	Cell counting and an MTT assay; Mineralization nodule counts, ALP activity, western blotting, qPCR (BSP, OCN, COL1a1), and immunohistochemistry	CGF promoted PDLSCs proliferation and osteogenic differentiation in a time- and dose-dependent manner.	
Rochira et al. (2020) [41]	h-BMSCs	Osteogenic differentiation	ALP activity, ARS, western blotting, qPCR (RUNX2, OSX, OPN, COL1a1)	CGF alone can induce osteogenic differentiation in h-BMSCs.	High RUNX2 expression and RUNX2 nuclear translocation are molecular mechanisms of h-BMSCs osteogenic differentiation induced by CGF.
Honda et al. (2013) [42]	hTERT-E6/E7 human MSCs	Proliferation, osteogenic differentiation	Cell counting; ALP activity, ARS, western blotting, qPCR (RUNX2, OSX, OPN, COL1a1)	CGF, at concentrations between 1 and 10%, promoted proliferation, osteogenic maturation, and mineralization of hTERT-E6/E7 human MSCs in a dose-dependent manner, and higher concentrations of CGF had an inhibitory effect.	

*Abbreviations*: *DPSCs* dental pulp stem cells. *SCAPs* stem cells of the apical papilla, *PDLCs* periodontal ligament cells, *PDLSCs* stem cells of the periodontal ligament, *BMSCs* bone marrow-derived mesenchymal stem cells, *MSCs* mesenchymal stem cells, *PDGF-BB* platelet-derived growth factor-BB, *bFGF* basic fibroblast growth factor, *TNF-α* tumour necrosis factor-α, *IL* interleukin, *TGF-β* transforming growth factor-β. *ARS* Alizarin Red S staining. *ALP* alkaline phosphatase. *MTT* 3-(4,5-dimethylthiazol-2-yl)-2,5-diphenyltetrazolium bromide, *DSPP* dentin saliva phosphoprotein, *DMP* dentin matrix protein, *COL1a* 1collagen I, *OCN* osteocalcin, *RUNX2* Runt-related homeobox2, *BSP* bone sialoprotein, *OPN* osteopontin, *OSX* osterix, *VEGFR2* vascular endothelial growth factor receptor 2, *CD31* cluster of differentiation 31, *SMAD* mothers against decapentaplegic homolog, *LPS* lipopolysaccharide

### Effects of CGF on SC proliferation and migration

CGF promotes DPC regeneration via a cell homing mechanism in which signalling molecules mediate the recruitment of endogenous cells such as stem/progenitor cells to the injured tissue [5]. This chemotactic effect of CGF on SCs is critical for tissue repair. It was previously demonstrated that CGF treatment enhanced the migratory capacity of DPSCs and PDLSCs, possibly via bFGF and the chemokine PDGF-BB [34, 37, 49]. The latter has the highest release concentration in CGF and was shown to promote the homing of dental pulp SCs [49]. bFGF, which has effects on DPSCs migration similar to granulocyte colony-stimulating factor in vitro, is also an effective homing/migration factor in pulp regeneration [50]. In one study, CGF increased the expression of the proinflammatory cytokine interleukin (IL)-8 in DPSCs, leading to the recruitment of tissue SCs to the site of injury [51]. Thus, PDGF-BB and bFGF may stimulate cell migration in part by promoting inflammation.

CGF is known to stimulate the proliferation of various MSC types (e.g., PDLSCs, DPSCs, and MSCs [hTERT-E6/E7]) in a dose-dependent manner, possibly through the independent or synergistic effects of GFs [36, 37, 40, 42]. However, some studies have reported a lack of dose dependence, which may be attributable to the different methods used to prepare CGF [34, 38]. Three methods for preparing CGF have been described to date—namely, spontaneous release into a medium [41], freeze-drying [47], and freeze-thawing [16]. The first two methods are often used in in vitro studies of CGF and yield highly variable extract variable concentrations. Highly concentrated CGF was shown to inhibit cell proliferation in some studies [38]; this effect is thought to be mediated by TGF-β and proteolytic enzymes in the preparations.

### Effects of CGF on SC differentiation

A key step in DPC regeneration is the differentiation of SCs into various cell types that crosstalk with surrounding cells [52]. The multidifferentiation potential of SCs meets the requirements of connective tissue formation, vascularisation, innervation, and dentin-like tissue deposition [53]. The generation of odontoblasts from SCs and dentin-like tissue deposition are essential for DPC regeneration and involve proliferation, cell aggregation, and ECM secretion and calcification [54]. Dentin saliva phosphoprotein (DSPP) and dentin matrix protein (DMP)-1, collagen I (COL1a1), alkaline phosphatase (ALP), and osteocalcin (OCN) have been used as osteogenic/odontoblastic differentiation-related markers [55, 56]. Among them, DSPP and DMP-1 are considered as odontoblastic differentiation-specific markers [57]. Accordingly, there is increasing interest in enhancing the efficiency of differentiation into odontoblasts/osteoblasts for pulp regeneration.

CGF has been shown to promote osteogenic/odontoblastic differentiation of DPSCs [37] and SCAPs [34] in vitro by inducing mineralised nodule formation and the expression of *COL1a1*, *ALP*, *OCN*, *DMP-1*, and *DSPP* genes, and osteogenic differentiation of PDLSCs [40] and BMSCs [41] by inducing the expression *COL1a1*, *ALP*, *OCN*, and *Osterix* (*OSX*) genes. In general, MSCs treated with CGF undergo osteogenic differentiation, but this is inhibited at high concentrations by proinflammatory factors such as tumour necrosis factor (TNF)-α and interleukin (IL)-1 in CGF extract, which may exert more potent effects than GFs [34, 42].

CGF promotes osteogenic/odontoblastic differentiation of SCs through the release of GFs including bFGF, BMP-2, and TGF-β1, which stimulate bone formation [58]. bFGF regulates mesenchymal condensation and is essential for cartilage formation, osteogenesis, and bone and mineral homeostasis in vivo [20]. TGF-β1 accelerates ECM synthesis in most physiological processes. BMP-2 plays a critical role in tooth development and promotes the terminal differentiation of odontoblasts [21]. Inhibiting any of these GFs suppresses the osteogenic differentiation of SCs [58].

GFs are known to act synergistically and their mechanisms of action involve the activation of Runt-related homeobox (RUNX)2, the main regulatory transcription factor in osteogenic/odontoblastic differentiation [59]. BMSCs and DPSCs cultured in CGF overexpress RUNX2 [36, 41]. BMP-2 promotes the expression of RUNX2 via the BMP-2/Mothers against decapentaplegic homolog (SMAD)5/Runx2 signalling axis in bone formation and remodelling, which is also involved in CGF-mediated DPSC mineralisation [36, 60]. The Wnt/β-catenin signalling pathway may also mediate the positive effect of CGF on osteogenic differentiation by activating the T cell factor (TCF)/lymphoid enhancer binding factor (LEF) transcription factor complex to induce RUNX2 expression [61]. It was reported that *Wnt3a* mRNA expression was increased in PDLSCs in a time-dependent manner by CGF treatment [62]. However, the component of CGF that activates the Wnt/β-catenin pathway remains to be identified.

### Effects of CGF on SCs in an inflammatory environment

Dental caries and trauma are associated with inflammation in the dental pulp tissue, which is difficult to control given the anatomy of the pulp cavity and can lead to pulp destruction and necrosis. It has been suggested that inflammation is a prerequisite for dental tissue healing, as low levels of proinflammatory factors trigger differentiation and mineralisation; on the other hand, high levels amplify the inflammatory response through the recruitment of more inflammatory cells [63]. One reason for the failure of vital pulp preservation and treatments is the difficulty of removing the infected pulp, which remains in a state of inflammation during persistent infection. Therefore, strategies to facilitate the repair of dental tissue in an inflammatory microenvironment to achieve pulp regeneration focus on current endodontic research.

It was reported that CGF can promote the proliferation, migration, and differentiation of DPSCs exposed to lipopolysaccharide (LPS) in vitro [35]. Pulp inflammation accompanying carious lesions is characterised by increased expression of TNF-α, IFN-γ, IL-1β, IL-6, and IL-18, which is induced in vitro in DPSCs by LPS, a toxic factor related to dental caries [64]. LPS stimulation was shown to promote the proliferation, migration, and differentiation of DPSCs [35]. CGF also plays a role in tissue repair by regulating the LPS-induced inflammatory response in DPSCs by inhibiting the expression of the proinflammatory cytokines IL-8 and TNF-α but not IL-6, which is thought to accelerate tissue repair by triggering the reprogramming of senescent cells [65]. TNF-α is known to suppress MSCs proliferation as well as osteogenic differentiation, which is induced by activation of TNF-α and nuclear factor (NF)-κB signalling and inhibition of RUNX2 expression [66]. CGF relieved the inhibitory effect of TNF-α on the osteogenic differentiation of SCs, which was related to the upregulation of *Runx2* transcription by GFs such as TGF-β1 in CGF [39]. However, although the above-mentioned in vitro studies indicate that CGF controls inflammation and promotes SC differentiation, the experimental conditions cannot fully mimic the in vivo local microenvironment of the DPC.

## Clinical application of CGF in DPC regeneration

**Fig. 3:**
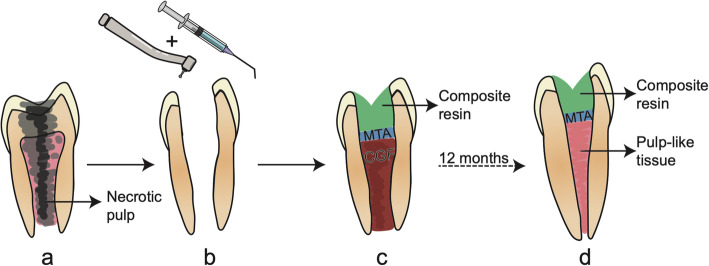
CGF used as root canal filling material in regenerative endodontic treatment. **a** An immature tooth with necrotic pulp. **b** Removal of decay lesion and necrotic pulp tissue. **c** CGF packed into the canals to the level of the cementoenamel junction and covered with and restored with composite resin. **d** After 12 months, pulp-like tissue formatted, root apex closure, and the thickness of the dentin increased

The use of CGF for DPC regeneration in clinical practice has yielded promising results. CGF is mainly used as root canal filling material to regenerate pulp tissue and pulp capping material to seal the pulp cavity. In vivo experiments have shown that when CGF was used as a scaffold in regenerative endodontic treatment, dental pulp-like tissue with blood vessels, nerves, and odontoblasts arranged in palisade formed in the root canal, and immature permanent teeth showed normal thickening of the root canal wall and apex closure [35]. The success rate of CGF combined with revascularisation in the treatment of apical hypoplasia in permanent teeth with dental pulp disease was 71.4% [67]; this is similar to the rate achieved with revascularisation therapy, which involves the filling of the root canal with blood to form blood clots, thus providing a microenvironment that is conducive to cell proliferation and odontogenic differentiation [68]. In one case study of a 21-year-old male patient with a fractured and discoloured non-vital maxillary left central incisor with an incompletely developed root and open apex, bleeding was induced and autologous CGF was packed into the canals to the level of the cementoenamel junction and covered with mineral trioxide aggregate; radiographic examination at the 12-month follow-up revealed that periapical lesions were reduced and the thickness of the dentin was increased [69]. Using a similar procedure, another study found that pulp vitality and sensory function were restored in the affected teeth [67]. In these cases, autologous CGF was an effective scaffold material that compensated for the absence of high-quality blood clots. However, a limitation of these reports is that there was no evidence that dentin DPC regeneration occurred. Randomised clinical trials with longer follow-ups are needed to confirm the efficacy of CGF for the regeneration of dentin DPC (Fig. 3).

**Fig. 4:**
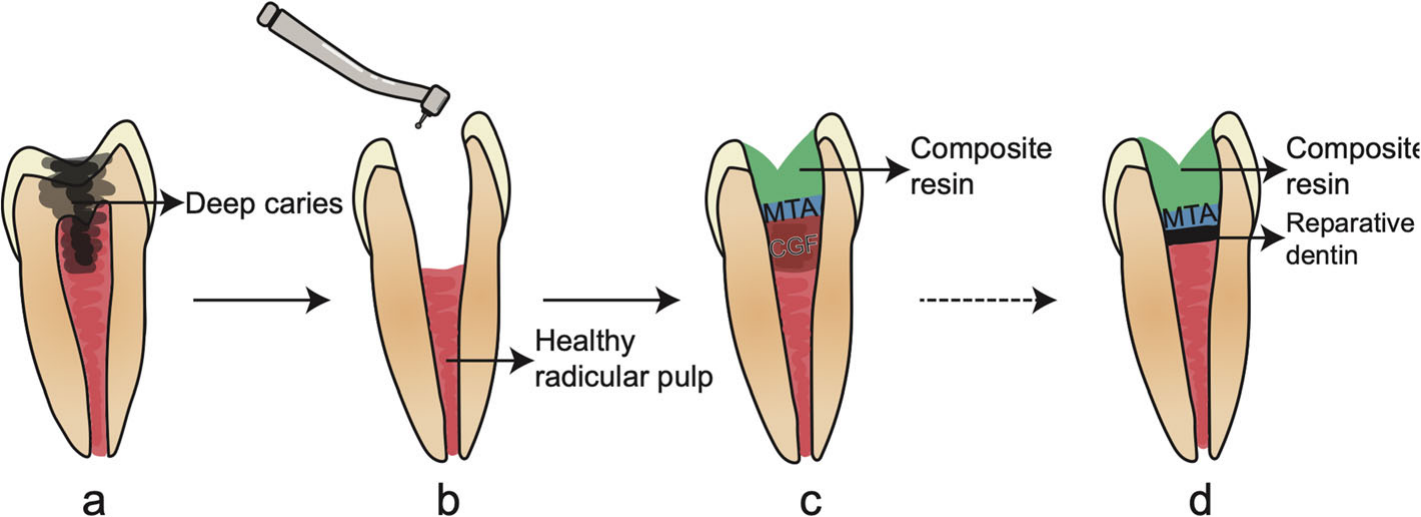
CGF used as pulp capping materials in vital pulp therapy. **a** A tooth with deep caries. **b** Removal of decay lesion and damaged coronal pulp tissue, and cavity preparation. **c** CGF placed on the remaining healthy radicular pulp tissue surface and covered with mineral trioxide aggregate (MTA) and restored with composite resin. **d** Reparative dentin formatted and preservation of the pulp health and vitality

Vital pulp therapy involves the application of pulp capping materials to promote the formation of a dentin bridge at the root canal orifice after removing the damaged coronal pulp tissue [70]. However, the severe inflammatory reaction caused by the material is a major reason for the failure of this treatment [71]. Basic experiments have proved that CGF can still promote the proliferation, migration, and differentiation of stem cells involved in the regeneration of DPC in the inflammatory microenvironment. In animal experiments, pulp capping with CGF gel resulted in the formation of a thin calcification barrier with odontoblasts in a regular arrangement on one side of the dentin bridge [36]. The regulation of the inflammatory response and induction of odontogenic SC differentiation by CGF could improve the long-term success rate of vital pulp therapy (Fig. 4).

## Conclusion

As the latest generation of platelet concentrate, CGF is superior to previous preparations in terms of composition and efficacy. CGF regulates the biological behaviour of dental SCs—especially in an inflammatory microenvironment—and is a therapeutic biomaterial that has been used successfully for endodontic treatment in a limited number of cases. Nonetheless, additional studies including randomised controlled clinical trials are needed to assess the clinical utility of CGF for DPC regeneration based on long-term outcomes.

## Data Availability

Not applicable.

## Abbreviations

DPC: Dentin–pulp complex
SCs: Stem cells
CGF: Concentrated growth factor
ECM: Extracellular matrix
RCT: Root canal treatment
GFs: Growth factors
PRP: Platelet-rich plasma
PRF: Platelet-rich fibrin
PPP: Plaletet poor plasma
RBC: Red blood cell
WP: White part
RP: Red portions
BC: Buffy coat
TGF-β1: Transforming growth factor-β1
PDGF-BB: Platelet-derived growth factor-BB
IGF-1: Insulin-like growth factor-1
BMP: Bone morphogenetic protein
VEGF: Vascular endothelial growth factor
EGF: Epidermal growth factor
bFGF: Basic fibroblast growth factor
DPSCs: Dental pulp stem cells
SCAPs: Stem cells of the apical papilla
PDLSCs: Stem cells of periodontal ligament
BMSCs: Bone marrow-derived mesenchymal stem cells
IL: Interleukin
DSPP: Dentin saliva phosphoprotein
DMP: Dentin matrix protein
COL1a: 1collagen I
ALP: Alkaline phosphatase
OCN: Osteocalcin
TNF: Tumour necrosis factor
RUNX2: Runt-related homeobox2
SMAD: Mothers against decapentaplegic homolog
TCF: T cell factor
LEF: Lymphoid enhancer binding factor
LPS: Lipopolysaccharide
NF: Nuclear factor
MTA: Mineral trioxide aggregate
